# Associations between early language, motor abilities, and later autism traits in infants with typical and elevated likelihood of autism

**DOI:** 10.1002/aur.3023

**Published:** 2023-09-12

**Authors:** Leyan Li, Greg Pasco, Jannath Begum Ali, Mark H. Johnson, Emily J. H. Jones, Tony Charman

**Affiliations:** ^1^ Department of Psychology Institute of Psychiatry, Psychology & Neuroscience, King's College London London UK; ^2^ Department of Psychological Sciences Centre for Brain and Cognitive Development, Birkbeck, University of London London UK; ^3^ Department of Psychology University of Cambridge Cambridge UK

**Keywords:** autism, language, motor ability, social responsive scale, standardized measures

## Abstract

Slower acquisition of language and motor milestones are common in infants with later autism and studies have indicated that motor skills predict the rate of language development, suggesting these domains of development may be interlinked. However, the inter‐relationships between the two domains over development and emerging autistic traits are not fully established. We studied language and motor development using standardized observational and parent‐report measures in infants with (*n* = 271) and without (*n* = 137) a family history of autism across four waves of data collection from 10 to 36 months. We used Random Intercept Cross‐Lagged Panel Models to examine contemporaneous and longitudinal associations between language and motor developments in both elevated and typical likelihood groups. We estimated paths between language and motor abilities at 10, 14, 24, and 36 months and autism trait scores at 36 months, to test whether the domains were interrelated and how they related to emerging autism traits. Results revealed consistent bidirectional Expressive Language (EL) and Fine Motor (FM) cross‐lagged effects from 10 to 24 and a unidirectional EL to FM effect from 24 to 36 months as well as significantly correlated random intercepts between Gross motor (GM) and Receptive language (RL), indicating stable concurrent associations over time. However, only the associations between GM and RL were associated with later autism traits. Early motor and language are linked, but only gross motor and receptive language are jointly associated with autistic traits in infants with an autism family history.

## Lay Summary

Children with autism sometimes show delays in language and motor skills from infancy. However, few researchers have focused on how language and motor skills develop together. We studied infants with a family history of autism over time, and found that early expressive language skills predicted later fine motor skills and vice versa. Gross motor (e.g., walking) and early language understanding predicted later autistic traits at age 3. Together, our results suggest that early motor and language are linked, but only gross motor and receptive language are jointly associated with autistic traits among children with autism family history.

## INTRODUCTION

Autism^1^ is a lifelong neurodevelopmental condition characterized by difficulties in social communication and interaction, the presence of restrictive and repetitive behaviors and interests and sensory anomalies (DSM‐5; APA, 2013). Although the etiology of autism is not fully understood, studies have shown it to have an estimated heritability of ~50%–90% (Bai et al., 2019; Gaugler et al., 2014; Tick et al., 2016). Infants who have older siblings or relatives with autism are more likely to be diagnosed with it themselves (Jones et al., 2014). In population studies, around 10% of children with a sibling with autism are autistic themselves (Jones & Szatmari, 2002; Szatmari et al., 2016); this rises to 20% in prospective cohorts (Messinger et al., 2015; Ozonoff et al., 2011) and can be up to 36% in children from multiplex families (i.e., children with more than one relative diagnosed with autism) (McDonald et al., 2020). Leveraging this heritability, prospective studies of infants with a family history of autism can provide insight into the early developmental changes that precede a later diagnosis.

Prospective designs can be used to identify early markers of autism as they enable researchers to use both subjective and objective measures to follow the developmental trajectories of cognitive abilities (Costanzo et al., 2015). Further, prospective design can provide insight into potential targets for pre‐emptive early intervention (Goldani et al., 2014; Wolff & Piven, 2021). Early intervention may have the potential to enhance individuals' communication and developmental abilities (Boyd et al., 2010; Dawson, 2008; Lord et al., 2020; Sandbank et al., 2020).

Two behavioral domains that are often delayed in young children with a diagnosis of autism are language and motor skills. Specifically, language delay affects the infant's opportunity to develop functional communication (Zager et al., 2012) and is thought to be closely associated with early emerging autism (Garrido et al., 2017). Longitudinal studies of early diagnosed autistic children indicate that whilst there is variability in the preschool period, from the age of 6 years language trajectories are relatively stable (Pickles et al., 2014). Similarly, delays in motor abilities are often seen in young children with a diagnosis (Fournier et al., 2010; Patterson et al., 2022) in domains including fine and gross motor abilities (Lloyd et al., 2013), oral and manual motor skills (Adams, 1998; Gernsbacher et al., 2008), and communicative gestures (Watson et al., 2013). Thus, studying the early emergence of both language and motor delays is important in understanding the early trajectories of autism.

Prospective studies of language and motor development in infancy show the gradual emergence of delays over developmental time. In the language domain, infants with an autistic sibling were reported as having significantly delayed receptive and expressive language abilities (Prelock & Nelson, 2012), and with larger receptive‐expressive differences than typically developed peers (Hudry et al., 2014; Mitchell et al., 2006), and in some studies as well as the fact that the receptive language has been found to be more impaired than expressive language and thus may be an important indicator of autism (Hudry et al., 2010). In the motor domain, prospective studies have shown that motor delays are common among infants with an autism family history, and it is more pronounced and persistent in those who later receive an autism diagnosis (LeBarton & Iverson, 2013). Similarly, delays in both fine and gross motor skills have been shown to be significantly associated with later autism (Lim et al., 2021), yet less is known about how and whether these domains are associated over development.

### The language‐motor link

In typically developing infants there are close associations between the development of motor and language abilities. The most common hypothesis for this association is the speech–motor integration theory, which proposes that the motor system (the premotor cortex and primary motor cortex), the functional hub of motor abilities, contributes to the perceptual processing and production of natural speech and complex vocal sounds (Hickok & Poeppel, 2007; Liberman et al., 1967; Skipper et al., 2017; Skipper & Hasson, 2017). This theory predicts prospective associations between motor skills and later language, such as motor performance at 3 months predicting language outcome at 2 years old (Peyton et al., 2018), and walking skills at 10 months predicting language development at 14 months (Libertus & Violi, 2016). In infants with a family history of autism, several behavioral studies using standardized or parental report‐based measures have also reported that motor ability predicts later language ability. For example, Gross motor skills at 2 years old predict subsequent Receptive and Expressive language abilities at age 9 years (Bedford et al., 2016), and Fine motor and overall motor abilities at both 1 and 2 years old predict Expressive language ability at age 3 years (LeBarton & Iverson, 2013; LeBarton & Landa, 2019; Leonard et al., 2015), as well as oral motor skills predicts speech and language development based on a longitudinal dataset with age ranging from 2 to 17 years old (Mody et al., 2017). However, these studies did not look at the reverse associations, or take into account concurrent language‐motor relations. Therefore, it is important to understand the trajectory of development of motor ability and its associations with receptive language/speech perceptual ability. It may be that by jointly examining both language and motor skills together over time potential markers of emerging autism could be revealed, contributing to the understanding of autism development.

### Current study

In the current prospective sibling study, we tested the developmental interrelation between language and motor skills between 6 months and 3 years in a prospectively assessed cohort of infants with (elevated likelihood) and without (typical likelihood) a family history of autism. To avoid associations based on the use of the same measurement scale, we used Mullen language and Vineland motor measures to construct structural equation models. We selected these measures as language development in young infants may be better assessed by direct observation, whilst milestones in motor development are more easily observed by parents in a broad range of contexts. Moreover, Mullen does not provide assessments of gross motor skills for up to 3 years.

We addressed the following questions: (1) How are motor and language abilities interrelated in infancy? (2) How does this interrelation differ in infants with and without a family history of autism? and (3) Is this interrelation associated with later autistic traits? We predict there will be a reciprocal predictive relation between motor and language development from 14 months (the age at which behavioral signs of autism become clearer (Jones et al., 2014), such that early language skills will predict later motor skills and vice versa, and that this relation will be stronger in the Elevated Likelihood group due to the accumulation of jointly emerging atypicality in both language and motor domains among children with later autism. Further, we also predict that both language and motor skills will jointly relate to later autism traits.

## METHOD

The study obtained ethical approval from the NHS National Research Ethics Service (08/H0718/76 and 06/13/LO/0751), and the Research Ethics Committee, Department of Psychological Sciences, Birkbeck, University of London.

### Participants

The dataset used in the present study is part of the ongoing British Autism Study of Infant Siblings (BASIS)/ Studying Autism and ADHD in the Early Years (STAARS) project (www.basisnetwork.org and https://www.staars.org/), a longitudinal prospective study of infants with and without a family history of autism and ADHD.

The present study included a total of 408 participants enrolled in the BASIS‐STAARS project including 271 Elevated Likelihood participants (46 participants with confirmed autism) and 137 Typical likelihood (TL) controls. Participants were recruited via the Birkbeck Centre for Brain and Cognitive Development volunteer database, community and online adverts and clinical networks.

Inclusion criteria included age (under 16 months); having a first‐degree relative (primarily older siblings but including parents) with a community clinical diagnosis of autism (Elevated Likelihood group) or older siblings with typical development (TL group); at least one parent who spoke English at home. Inclusion criteria included full‐term birth (gestational age > 36 weeks) and no known medical or developmental condition (Further details in Supplementary Information).

### Measures

#### Language assessment

The Mullen Scales of Early Learning (Mullen, 1995) was administrated by researchers at 10, 14, 24, and 36 months, providing both receptive (RL) and expressive language (EL) sub‐scales.

#### Motor skills

The Vineland Adaptive Behavior Scales, 2nd edition (Sparrow et al., 2005), was also administered at 10, 14, 24, and 36 months, conducted via the Survey Parent/Caregiver report (at 10 and 14 months) and Interview Form at 2 and 3 years by a trained researcher, providing both fine (FM) and gross motor (GM) scores.

#### Autism traits

The Social Responsiveness Scale, Second Edition (SRS‐2) (Constantino & Gruber, 2012) was completed by parents at 36 months and we use the SRS‐2 Total raw score as a measure of autism traits.

### Analytic plan

We used structural equation models separating within‐and between‐person effects, the Random Intercept Cross‐Lagged Panel Model (RI‐CLPM; Hamaker et al., 2015), to test if trajectories of language and motor abilities are closely associated/co‐developed, and if this co‐development is specific to the Elevated Likelihood group only, and whether language and motor skills jointly predict emerging autistic traits; for a successful analysis using RI‐CLPM see Baribeau et al. (2022).

We initially used Pearson correlations to explore whether there are bivariate correlations between sub‐scales of both language and motor abilities (at 10, 14, 24, and 36 months) and the 36 months SRS total score, as well as the correlations between the same subscales from different measures and SRS total scores to examine cross measure validity and associations. Then, Confirmatory factor analysis was implemented to assess the validity of hypothesized latent factors Language and Motor constituted by subscale scores of Mullen and Vineland scales, respectively.

We then used four multi‐group outcome RI‐CLPMs (i.e., Vineland FM & Mullen EL, Vineland GM & Mullen RL) regressing in 36 months SRS total scores as the outcome variable to assess how observed values of language and motor abilities predict autism trait values in both groups. Then, to test if the group variables contribute to the difference in paths between groups, we used a post‐hoc ANOVA to compare the degree of freedom and chi‐square statistics from both a multi‐group model and a full‐constrained model (group‐loading set to be equal across groups). If a significant difference was found between the two models, this suggests autism likelihood does affect paths. For detail about the functions of RI‐CLPM see (Mulder & Hamaker, 2021).

Models were estimated using maximum likelihood to account for missing data. Model fit was assessed by comparative fit index (CFI), root means of square error of approximation (RMSEA) and standardized root mean square residuals (SRMR). According to the developer of RI‐CLPM, an acceptable fit is indicated by CFI >0.90, RMSEA <0.08, SRMR <0.08, whereas a good fit is indicated by CFI >0.95, RMSEA <0.05, SRMR <0.05 (Hamaker et al., 2015). Models were estimated using observed variances via the lavaan package 0.6–10 in Rstudio‐2022.02.

## RESULTS

### Preliminary analyses

Sample characteristics, missing data patterns for all measures and likelihood groups are shown in Table 1 and Supplementary Table 1. Participants in the Elevated Likelihood group had significantly lower language scores (EL & RL at 24 & 36 months), lower fine motor scores at all timepoints and significantly higher SRS scores at 36 months than the Typical Likelihood group. Similarly, those receiving a later autism diagnosis had significantly lower language scores (EL at 14, 24, and 36 months, RL at all timepoints), and lower gross motor scores at 36 months, but no difference in fine motor scores at all timepoints.

**TABLE 1:** Sample characteristics, descriptive statistics by likelihood groups and independent sample *t*‐tests.

Logistic parameter		10 months				14 months				24 months				36 months			
		*M*	SD	*p*	Cohen's d	*M*	SD	*p*	Cohen's d	*M*	SD	*p*	Cohen's d	*M*	SD	*p*	Cohen's d
Vineland Fine motor	EL	10.90	3.83	0.001***	0.350	16.84	3.78	0.001***	0.371	23.30	5.51	0.028*	0.249	32.71	7.28	0.001***	0.547
	TL	12.18	3.26			18.18	3.14			26.03	4.73			36.78	7.77		
	EL‐not Autism	11.25	3.90	0.133	0.250	16.83	3.75	0.654	0.074	25.06	4.98	0.092	0.283	32.99	6.76	0.052	0.326
	EL‐Autism	10.27	3.99			16.56	3.72			23.63	5.40			30.72	7.94		
Vineland Gross motor	EL	13.89	6.83	0.024*	0.251	34.08	9.84	0.546	0.068	53.78	6.41	0.113	0.179	61.70	6.76	0.002**	0.362
	TL	15.63	7.12			34.75	9.34			54.87	5.41			64.04	5.60		
	EL‐not Autism	14.86	7.27	0.069	0.303	34.21	9.68	0.809	0.040	54.13	6.33	0.097	0.279	62.40	6.08	0.001***	0.640
	EL‐Autism	12.66	7.30			33.82	10.47			52.37	6.15			58.28	7.90		
Mullen Expressive language	EL	9.02	2.66	0.747	0.033	13.17	3.50	0.053	0.202	23.30	5.51	0.001***	0.399	33.63	6.83	0.001***	0.584
	TL	8.97	2.31			13.84	3.02			25.38	4.62			37.29	4.87		
	EL‐not Autism	9.03	2.67	0.861	0.029	13.48	3.43	0.001***	0.527	23.86	5.36	0.001***	0.574	34.71	5.93	0.001***	0.858
	EL‐Autism	8.96	2.56			11.67	3.43			20.74	5.72			29.23	8.36		
Mullen Receptive language	EL	9.40	2.23	0.326	0.105	13.58	3.24	0.056	0.210	25.09	5.00	0.001***	0.457	33.62	6.06	0.001***	0.613
	TL	9.65	2.38			14.28	3.47			27.19	3.63			37.07	4.59		
	EL‐not Autism	9.89	2.24	0.004**	0.471	13.86	3.31	0.034*	0.346	25.95	4.44	0.001***	1.073	34.71	5.06	0.001***	0.848
	EL‐Autism	8.80	2.63			12.76	2.31			20.95	5.58			29.84	8.34		
SRS‐2 total raw score	EL													42.37	33.73	0.001***	0.622
	TL													24.48	14.93		
	EL‐not Autism													32.71	23.30	0.001***	1.914
	EL‐Autism													83.80	39.27		

*Note*: N_EL_ = 271, N_TL_ = 137, N_EL‐not Autism_ = 223, and N_EL‐Autism_ = 46. Bold *p*‐values represents *p* < .05.

Abbreviations: EL, elevated likelihood; Mullen, Mullen Scales of Early Learning Scale; SRS‐2, Social Responsiveness Scale, Second Edition; TL, typical Likelihood; Vineland, Vineland Adaptive Behavior Scales Third Edition.

Language and motor scores (sub‐scales) and the same type of subscales from different measures (i.e., Vineland language and Mullen language) were generally correlated from 10 to 36 months across all groups, and correlations between the longitudinal language and motor factors (including across the different measures) and SRS total raw score were all significant from 14 to 36 months (see Supplementary Tables 2, 3 and 4).

Confirmatory factor analyses were used to test if the assumed sub‐scale language and motor latent factors are supported by the longitudinal observed variables including four indicators FM, GM, EL, and RL. Results suggested the latent factors of language and motor scores were estimated with overall good model fits with the present dataset in the converged models (For detail see Table 2 and Supplementary Figure 1).

**TABLE 2:** Developmental cascade model of language and motor in first three years—fit criteria and model selection.

Model	AIC	Chi^2^, DF	CFI	SRMR	RMSEA (UL LL)	ANOVA p	*R* ^2^ of outcome
Criteria for acceptable fit	Smaller is better		>0.90	<0.06	<0.08		
Confirmatory factor analysis							
CFA for model 1	16,294	698.54, 28	1.00	0.00	0.00 (0.00, 0.00)		
CFA for model 2	17,283	668.04, 28	1.00	0.00	0.00 (0.00, 0.00)		
Random‐Intercept CLPM							
1. FM & EL Multi‐group outcome	**16,956**	**14.90, 18**	**1.00**	**0.02**	**0.00 (0.00, 0.05)**	***p* = 0.031** *	**0.117**
2. GM & RL Multi‐group outcome	**17,930**	**24.89, 18**	**0.99**	**0.02**	**0.04 (0.00, 0.08)**	**NA**	**0.467**

*Note*: Bolded values indicate model fits significantly changed due to group variables; NA indicated that the baseline (constrained) model did not converge, suggesting the basic model fits significantly better.

Abbreviations: ANOVA p, *p*‐value of ANOVA test between basic multi‐group model and constrained model; CFI, Comparative Fit Index; CLPM, Cross‐Lagged Panel Model; RMSEA, Root Mean Square Error of Approximation; SRMR, Standardized Root Mean Square Residual.

* *p* < .05.

### Data transformation for the SRS score at 36 months

As the SRS Total score was strongly positively skewed and leptokurtic (a distribution with thick tails), we implemented a cube root data transformation to both ensure the model fit of the structural equation model and to maintain existing associations between the target and observed variables (Bentler & Bonett, 1980; West et al., 1995). The SRS scores reached approximately normal and mesokurtic distribution after cube root transformation (Original data: Skewness = 1.820, Kurtosis = 5.837; Transformed data: Skewness = 0.815, Kurtosis = 3.338; Standard for Skewness is 0 and for Kurtosis is 3), and the post‐hoc validating linear model suggested significances are maintained within the current dataset (for comparison and plots of transformed data, please see Supplementary Figure 2).

### Multi‐group RI‐CLPM with SRS scores to explore relationships between hypothesized language and motor co‐development and later autistic traits

To test whether features of language‐motor co‐development are early markers of later autistic traits, the SRS scores were regressed on both Random intercepts and latent variables, as the outcome variable, in the four multi‐group outcome RI‐CLPM models (FM & EL, FM & RL, GM & EL, and GM & RL).

**FIGURE 1:**
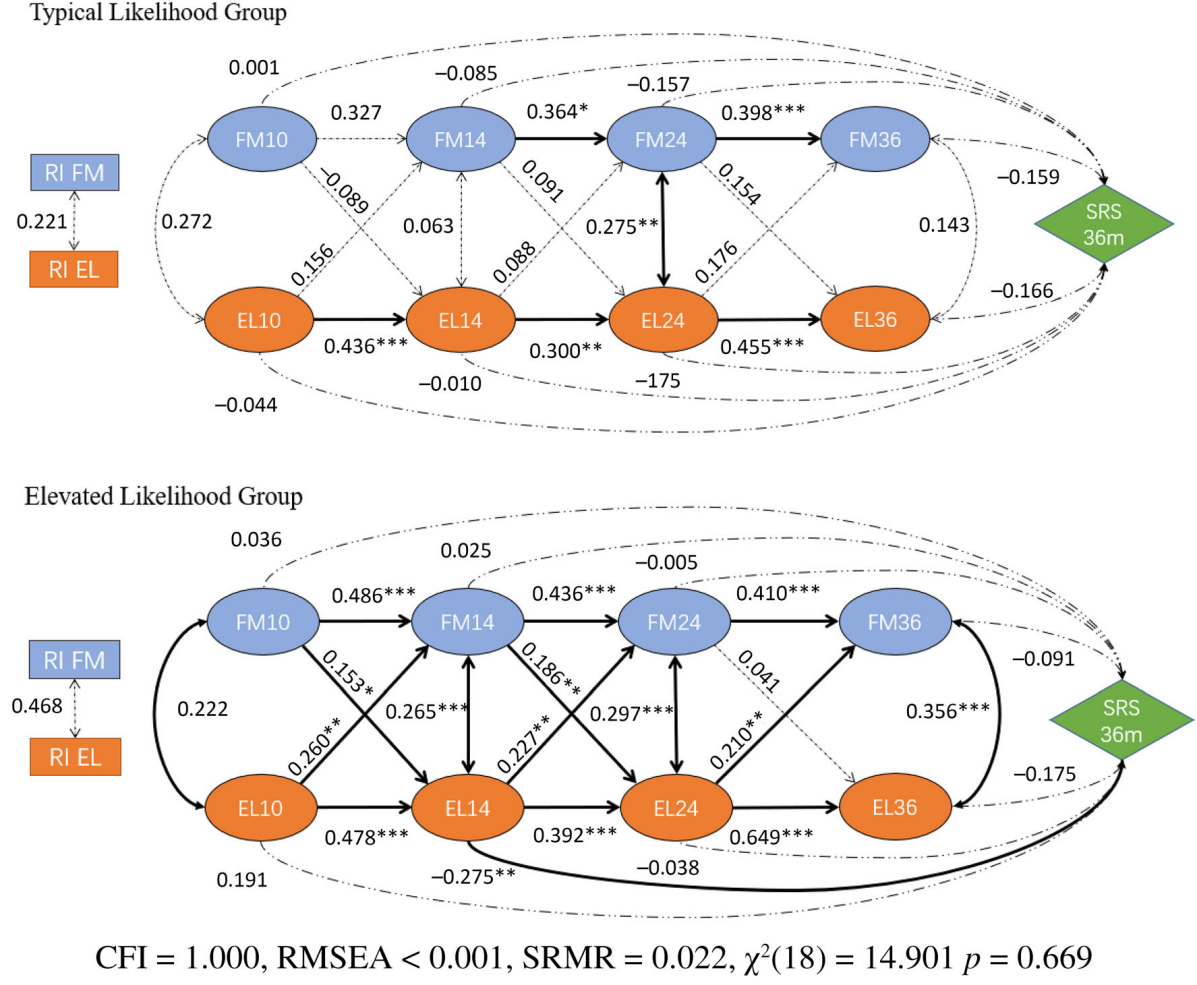
Structure of a multi‐group random intercept cross‐lagged panel model estimated with Vineland fine motor and Mullen expressive language sub‐scales and regressed with SRS scores at 36 months as the outcome variable. Values in the figure indicate a standardized coefficient, *** indicates a significance level at 0.001 and bold paths indicate significant effects.

As none of the models with SRS regressed on random intercepts of language and motor subscales converged with acceptable model fits, we only report models with SRS scores regressed on latent variables (Figure 1).

#### Fine motor (FM) and expressive language (EL)

The Baseline ANOVA test suggested a significant advance on the basic model (*p* = 0.031; Table 2) and the fully constrained model showed a significantly poorer fit, hence supporting the Likelihood group variable's contribution to the difference in paths between groups. The Fine Motor and Expressive Language‐SRS outcome RI‐CLPM model was estimated with a good fit (*χ* ^2^(18) = 14.901, *p* = 0.669; CFI = 1.000, RMSEA <0.001, SRMR = 0.022). The result indicates that it is only in the Elevated Likelihood group that there are significant bidirectional cross‐lagged paths between expressive language and fine motor abilities (from Language to Motor: *β*_10‐14_ = 0.260, *p*_10‐14_ = 0.005; *β*_14‐24_ = 0.227, *p*_14‐24_ = 0.002; *β*_24‐36_ = 0.210, *p*_24‐36_ = 0.002. from M to L: *β*_10‐14_ = 0.158, *p*_*10‐14*_ = 0.035; *β*_14‐24_ = 0.186, *p*_14‐24_ = 0.010), and the SRS 36 months score is also negatively associated with expressive language at 14 months (*β*_SRS 36m_ = −0.275, *p*_SRS 36m_ = 0.002).

**FIGURE 2:**
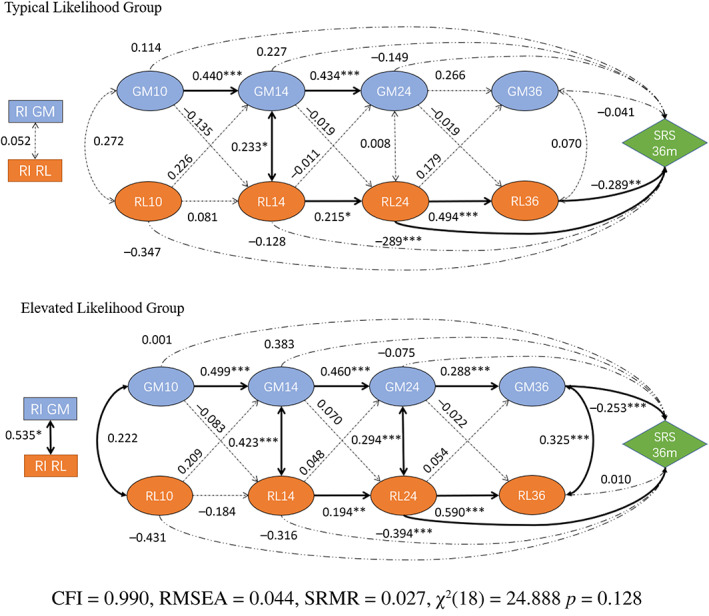
Structure of a multi‐group random intercept cross‐lagged panel model estimated with Vineland gross motor and Mullen receptive language sub‐scales and regressed with SRS scores at 36 months as the outcome variable. Values in the figure indicate a standardized coefficient, *** indicates a significance level at 0.001 and bold paths indicate significant effects.

To test whether children with an autism diagnosis fully explained these observations, we re‐ran the main models without children with an autism outcome. The full model including SRS traits with the autism cases removed did not converge; a model without SRS traits and with the autism cases removed replicated most of the cross‐lagged effects except those between 10 and 14 months **(**Supplementary Figure 3). We then implemented two sets of linear regressions between identified correlated latent factors and the autism trait score to determine whether results were wholly driven by children with an autism outcome. The results showed that the regression model including 14 month Expressive language as a predictor and SRS total scores as an outcome was significant with (*R* ^2^ = 0.074 (*p* < 0.001)) and without the EL‐Autism cases (*R* ^2^ = 0.029 (*p* = 0.026)), but the latter model had a lower *R* ^2^ (for detail see Supplementary Table 6); hence, confirming the association between 14 month Expressive language and the SRS total score not wholly driven by autism cases (Figure 2).

#### Gross motor (GM) and receptive language (RL)

The constrained GM and EL model did not converge, even when iterations were increased to 1 × 10^5^. The basic unconstrained model, therefore, fitted better, indicating that the group variable contributed to differences in paths between groups. The GM & RL_SRS outcome RI‐CLPM model is estimated with a good fit (*χ* ^2^(18) = 24.888, *p* = 0.128; CFI = 0.990, RMSEA = 0.044, and SRMR = 0.027). The result indicates that the SRS total score at 36 months is negatively associated with the receptive language ability at 24 and 36 months in the TL group (*β*_SRS‐RL 24_ = −0.289, *p*_*SRS‐RL 24*_ = 0.010; *β*_SRS‐RL 36_ = −0.289, and *p*_SRS‐RL 36_ = 0.005), whilst in Elevated Likelihood group the SRS score is negatively correlated with both receptive language and gross motor abilities at 24 and 36 months, respectively (*β*_SRS‐RL 24_ = −0.394, *p*_SRS‐RL 24_ < 0.001; *β*_SRS‐GM 36_ = 0.210, and *p*_SRS‐GM 36_ = 0.001). Moreover, the Elevated Likelihood group model includes a significant association between random intercepts of gross motor and receptive ability (*β*_RI‐GM & RI‐RL_ = 0.535 and *p*_RI‐GM & RI‐RL_ = 0.015). We then implemented similar steps using GM and RL as in the above FM‐EL model to test whether effects were driven by autism cases. The model with autism cases excluded did not converge. Results of substitute regression showed that both the 24 months Receptive language and 36 months Gross motor significantly regressed on‐SRS total score, though the *R* ^2^ the for total Elevated likelihood group dropped from *R* ^2^ = 0.213 (*p* < 0.001) to *R* ^2^ = 0.084 (*p* < 0.001) when the EL‐Autism cases were excluded (for detail see Supplementary Table 7); therefore, confirming both identified predictive relations was not fully driven by autism cases.

#### Gross motor (GM) and expressive language (EL)

This model did not converge as a multigroup model or as a constrained model with SRS traits. However, a uni‐group model omitting the outcome SRS scores did converge (GM & EL: CFI = 0.990, RMSEA = 0.045, SRMR=. 025) and found 10‐months EL predicts 14‐month GM (for details in Supplementary Figure 5). Moreover, multiple‐group RL‐CLPMs without SRS scores also converged with a good fit, but the unconstrained model did not significantly differ from the constrained model in a post hoc ANOVA (GM & EL: post‐hoc ANOVA *p*‐value = 0.897). In summary, these results suggest that neither autism traits nor autism likelihood group related to gross motor and expressive language associations.

#### Fine motor (FM) and receptive language (RL)

The FM & RL outcome model did not converge, but a uni‐group FM&RL model without SRS traits did converge with a good fit (FM & RL: CFI = 0.990, RMSEA = 0.025 SRMR=0.42) and suggested RL at 14 and 24 months predicts FM at 24 and 36 months (Supplementary Figure 6). Moreover, multiple‐group RL‐CLPMs without SRS scores also converged with a good fit, but the unconstrained model did not significantly differ from the constrained model in post hoc ANOVA tests (FM&RL: post‐hoc ANOVA *p*‐value = 0.538). In summary, these results suggest that neither autism traits nor autism likelihood group related to fine motor and receptive language associations.

## DISCUSSION

In the present study, we used a prospective design to examine the relationship between language and motor scores from infancy, and their relations to later autistic traits. We observed reciprocal cross‐lagged effects between fine motor and expressive language indicating extensive interdomain links in the Elevated Likelihood group only. In addition, random intercepts of gross motor and receptive language were also found to be significantly correlated in only the Elevated Likelihood group, suggesting concurrent associations between the two domains related to autism family history. Within Gross motor & Expressive language and Fine motor & Receptive models, there was no evidence for differences in the relation between language and motor domains by likelihood group. In terms of autism trait scores, results indicated expressive language at 14 months, receptive language at 24 and 36 months and gross motor skills at 36 months were negatively associated with 36‐month autism traits, suggesting complex associations between infant language and motor abilities and later autism traits.

### Likelihood group differences in both language and motor domains

Language and motor abilities are thought to be delayed in infants with a family history of autism. At a group level, language and motor skills in the Elevated Likelihood group were lower than in controls in the present sample. We also identify significant delays in Fine motor from 10 months and gross motor measures at 10 and 36 months in the Elevated likelihood infants compared to the Typical likelihood controls. This extends findings and is partially consistent with a review summarizing developmental differences in language and motor between infants with and without family history of autism based on various standardized measures (Vineland, Mullen, etc.) of Garrido et al. (2017) who reported that Fine motor skills are delayed at 12, 24, and 36 months and Gross motor skills delayed at 10 months only. We also observe significant group differences in Expressive language ability at 24 and 36 months, aligning with Garrido et al. (2017) who observed group differences from 12 to 36 months. Moreover, we observed significant group differences in Receptive language from 24 months, yet Garrido et al. (2017) found delays at 12, 24, and 36 months. Some of the minor discrepancies with Garrido et al. (2017) may be due to language and motor ability scores being assessed based on various types of measures and various populations around the world, as well as the fact that this systematic review lacks gross motor studies at 24 and 36 months. Further, as expected, infants with a family history of autism showed more autistic traits than infants without a family history of autism (*p* < 0.001, Cohen's *d* = 0.622, Table 1).

### Associations between fine motor skills and expressive language

In previous work, fine motor and expressive language abilities were frequently reported to be associated within Elevated Likelihood groups, and the fine motor delays occurred earlier than expressive language delays, leading to the hypothesis that early delayed fine motor contributed to later slowly developed expressive language ability (LeBarton & Iverson, 2013). However, in the present study, we found bidirectional cross‐lagged effects between 10 to 24 months with both forward prediction and concurrent relations between both expressive language and fine motor domains, unlike our initial hypothesis that there would be a reciprocal predictive relation between language and motor from 14 months. This suggests that later language difficulties are more closely related to slower development of both language and motor abilities associated with autism family history, rather than being caused specifically by delayed fine motor skills. There may also be a developmental cascade in which both motor and language skills reinforce each other over time, with advanced motor skills providing more opportunities for the child to learn language and advanced language skills allowing the child access to new motor learning opportunities, which is consistent with proposals by Leonard et al. (2015) and Gonzalez et al. (2019) who found language and motor development are both bi‐directionally predictive and cross‐sectionally linked among autistic individuals and the Elevated Likelihood group. Similarly, our findings do support a language‐motor‐specific development process rather than a more global developmental association. This is due to the facts that both (1) Visual Reception does not have a similar relation to both language and motor skills as patterns of cross‐lagged associations between FM‐EL and FM‐VR and are more variable across time, and (2) the patterns of FM‐EL in both models are highly overlapped except the path from 24 EL to 36 FM (see Supplementary Figure 4).

Notably, the only predictive relation to later autistic traits observed in this model was between expressive language at 14 months and later SRS scores. Interestingly, 14 months is the age at which behavioral symptoms of autism often become clearer (Jones et al., 2014). However, it is perhaps surprising that later measured expressive language and fine motor abilities did not relate to autistic traits. This is not consistent with previous studies which suggested delays in FM became more severe from 24 months onwards and delays in expressive language start from 24 months onwards in the EL group We had previously considered the possibility that the occurrence of joint delays in the elevated likelihood group is driven by a subgroup of participants who later receive autism diagnoses as they may have emerging impairment in both domains indicated by high SRS raw score. However, the lack of association between fine motor and expressive language skills and later autistic traits is not consistent with this. We examined our models whilst omitting participants with a later diagnosis; these models failed to converge, potentially consistent with these participants with autism outcomes providing important variance in the dataset. We observed that patterns of bivariate associations did not change significantly with participants with an autism outcome excluded, indicating that this failure to converge was unlikely to be related to significant changes in the pattern of association between motor and language skills. To confirm this, we ran a random intercept cross‐lagged panel model on fine motor and expressive language scores without the inclusion of SRS scores and with the participants with autism outcome excluded. The results indicated paths from 14 months onwards were not altered significantly; the bi‐directional 10–14 month paths were no longer significant **(**Supplementary Figure 3). This is similar to results of linear regression models of the 14 month expressive language and later SRS total scores that showed that the explained variances dropped but remained significant when taking out autism cases **(**Supplementary Table 6). Therefore, the results above raise the intriguing possibility that earlier associations between language and motor skills may be more related to autism diagnosis than later associations, which may extend to the broader cohort. Therefore, even though associations between delayed fine motor and expressive language abilities were found in only the Elevated Likelihood group, we did not find clear evidence that the later delays were necessarily solely related to autism outcomes.

The language‐to‐motor development contribution we demonstrate here differs from that found in a growth‐curve modeling study (of a partially overlapping dataset) where it was suggested that abilities in one domain did not affect the rate of change in other domains, in terms of coupling of language and motor abilities (Deserno et al., 2023). There are several differences between the two studies; Deserno et al. used scales from the same assessment in which subscales are unusually correlated with each other and confounded by shared methods variance, which might affect the results of their relationship. Moreover, Deserno et al. also used a different modeling structure, in which they were testing whether the rate of change in one skill predicts another, whereas we are testing whether individual differences at one timepoint relative to an individual's mean ability predict individual differences in another domain at a later point.

### Concurrent associations between gross motor and receptive language, and its relation to autism traits

Gross motor skills are also thought to be closely related to, or even predictive of, the development of language comprehension and production in typically developing infants, which has important implications for autism (Bedford et al., 2016; Garrido et al., 2017). However, only one study to date reported that walking (one milestone of gross motor development) significantly predicted the development of receptive language ability from 2 to 9 years (Bedford et al., 2016), suggesting gross motor changes in the rate of receptive language development among Elevated Likelihood infants. However, the results of our modeling suggested no consistent Gross motor‐Receptive language cross‐lagged effects. Rather, Gross motor and Receptive language stably covaried throughout development, reflected in positively associated random intercepts between gross motor and receptive language. This indicates a bi‐directional reciprocal effect that, averagely, participants with higher (lower) scores in one domain (i.e., gross motor) would have higher (lower) scores in the other domain (i.e., receptive language) (Mulder & Hamaker, 2021), suggesting strongly covaried concurrent associations in between the development of the two cognitive abilities.

The pattern of concurrent Gross motor and Receptive language associations is consistent with previous research in which motor brain regions were consistently activated during passive listening in adverse conditions and hence was believed to assist speech perception and language processing (Du et al., 2014; Stokes et al., 2019). Indeed, in the early developmental stage, infants explore their surroundings by crawling and walking (gross motor abilities) to increase their opportunities to contact information (i.e., sound and speech) (Walle & Campos, 2014). It is therefore not surprising that gross motor and receptive language abilities co‐varied due to the Gross motor‐Receptive language interrelation along with the development of autistic individuals (Wu et al., 2021). Another possibility is that these effects were also driven by a subgroup with emerging autism as we found these models no longer converged when autism cases were removed; this subgroup may have contributed to strong concurrent associations between Gross motor and Receptive language skills as well as between these skills and autism traits. Examination of the raw data indicated that many of the participants with autism had low Gross motor and Receptive language scores at 24 and particularly 36 months of age. Hence, exclusion of these participants reduced both the overall sample size and the variance in the measures, particularly at later timepoints, confirmed by the linear regressions that showed that the variances explained in regression dropped significantly when taking out the autism cases (Supplementary Table 7). Therefore, those concurrent associations are partially but not fully driven by the children with autism.

Regarding the relation between Gross motor and Receptive language and autism traits, in our modeling which explained a high percentage of the outcome variance (SRS raw scores) (*R* ^2^ = 0.467), we identified both 24 months Receptive language and 36 months Gross motor ability as being significantly negatively associated with the 36 months SRS raw score in the Elevated likelihood group. Thus, infants in the Elevated Likelihood group with lower 24 months Receptive language and 36 months Gross motor scores tended to have more elevated autistic symptoms at age 3 years. In contrast, in the Typtical likelihood group later SRS traits score were predicted by 24 and 36 months receptive language ability. We believe that these associations were found because individuals who exhibited exceptionally high Receptive language scores at 24 and 36 months will also develop typically (low SRS raw scores at 36 months) since the SRS tends to pick up the developmental skills as well as atypicalities at this young age of 3 years. Furthermore, the lack of association between Gross motor skills at 36 months and SRS total score in the Typical likelihood group, unlike the Elevated likelihood group, is likely attributable to the relatively limited variability within the group, as well as the absence of participants with severely delayed Gross motor abilities in our sample.

### Other domains

Models failed to converge between Gross motor and Expressive language and in between Fine motor and Receptive language when autism likelihood (grouping variables) and 36 months autism traits score (outcome variables) were included. When modeling data without autism measures, we did find strong associations between the two pairs without these constraints, such as that the Receptive language ability at 14 and 24 months predicts Fine motor ability at 24 and 36 months, and the Expressive language ability at 10 months predicts 14‐month Gross motor ability. This suggested predictive patterns were present but were not related to autism traits or autism family history, indicating the general language‐motor developmental associations among all participants as it has been frequently reported in typically developing infants (Hickok & Poeppel, 2007; Iverson, 2010).

### Strengths and limitations

The main strengths of the present study are the prospective longitudinal sibling design, where all participants are followed from 10 to 36 months, capturing a comprehensive picture of dynamic developmental trajectories, and the comparatively large sample size. We extended previous findings on language and motor development in autistic children and Elevated Likelihood infants as we identified a clear developmental pattern of language and motor development based on evidence of stable associations between Gross motor and Receptive language, and the bidirectional cross‐lagged association between Fine motor and Expressive language via the novel RI‐CLPM, as well as identifying language's unique contribution to later motor development.

However, we acknowledge some limitations. First, our sample size is not large enough to support more complex models. Future research should use larger cohorts to estimate models with outcome variables directly regressed on random intercepts so as to answer if language‐motor co‐development predicts later autism outcomes. Second, in the current study, we used two types of standardized measures for language and motor abilities, and the SRS and Vineland are both based on parental reports and are thus liable to shared method variance. However, the correlations between variables based on these measures (Supplementary Table 3) indicated that the SRS total score was equally associated with identified Mullen (observational scale) and Vineland (parental report) scores and their opposite scores, so shared methods variance does not appear to account for all the effects found. Finally, we acknowledge that the effect size of the Fine motor and Expressive language association we find is modest (*R* ^2^ = 0.117), suggesting only moderate effects in association between the two latent factors in terms of variances explained.

## CONCLUSION

The present study found that language and motor abilities measured by standardized scores were concurrently associated within the Elevated Likelihood group from 10 to 36 months. Specifically, we found both concurrent and predictive bi‐directional associations between fine motor scores and expressive language scores in the Elevated Likelihood group only; we also found positively co‐varied random intercepts of receptive language and gross motor abilities in the Elevated Likelihood group. Moreover, autistic traits at age 3 were independently predicted by Expressive language at 14 months, Receptive language at 24 months and Gross motor at 36 months in the elevated likelihood group. Based on this evidence we suggest that the jointly delayed receptive language skills and gross motor ability are relevant to indicating emerging autism.

### Clinical implications

Findings of the current study indicate that language and motor abilities, as well as motor‐language joint delays, can be used to identify infants who may later go on to autism. This strengthens the case for intervention in the developing language and motor systems in infancy, and as a result, potentially improving broader social communication and behavioral outcomes in infants at elevated likelihood for autism.

## AUTHOR CONTRIBUTIONS

Prof Charman, Prof Jones, and Prof Johnson obtained funding, conceived and designed the study, and were involved in the interpretation of data, and drafting and critical revision of the manuscript for important intellectual content. Mr Li had full access to all of the data in the study and takes responsibility for the integrity of the data and the accuracy of the data analysis, and was involved in the interpretation of data, and drafting and critical revision of the manuscript for important intellectual content. Dr Pasco and Dr Begum Ali were involved in the interpretation of data and drafting and critical revision of the manuscript for important intellectual content. All authors approved the final manuscript as submitted and agree to be accountable for all aspects of the work.

## FUNDING INFORMATION

This research received funding from the UK Medical Research Council (G0701484, MR/K021389/1, and MR/T003057/1) and the BASIS funding consortium led by Autistica, and Autism Speaks. Prof Mark Johnson, Prof Emily Jones and Prof Tony Charman were supported by the Innovative Medicines Initiative joint undertaking grant agreement no. 115300 (EU‐AIMS), resources of which are composed of financial contributions from the European Union's Seventh Framework Programme (FP7/2007–2013) and EFPIA companies' in‐kind contribution) and the Innovative Medicines Initiative 2 Joint Undertaking (IMI 2 JU) under grant agreement no. 777394 (AIMS‐2‐TRAILS). This Joint Undertaking receives support from the European Union's Horizon 2020 research and innovation program, EFPIA, Autism Speaks, Autistica, and SFARI. The funders had no role in the design of the study; in the collection, analyses, or interpretation of data; in the writing of the manuscript, or in the decision to publish the results. Any views expressed are those of the authors and not necessarily those of the funders (IHI‐JU2). Prof Jones and Charman were supported by Horizon Europe grant no. 101057385 (R2D2‐MH) and from UK Research and Innovation (UKRI) under the UK government’s Horizon Europe funding guarantee grant no.10039383. Capital equipment funding from the Maudsley Charity (980) and Guy's and St Thomas' Charity (STR130505). LL is supported by King's‐China Scholarship Council PhD Scholarship and received a grant from Henry Lester Trust.

## CONFLICT OF INTEREST STATEMENT

Prof Charman has served as a paid consultant to F. Hoffmann‐La Roche Ltd. and Servier; and has received royalties from Sage Publications and Guilford Publications. Prof Johnson has received royalties from OUP, MIT Press, and Wiley‐Blackwell. The other authors have no relevant conflicts to disclose.

## ETHICS STATEMENT

The study obtained ethical approval from the NHS National Research Ethics Service (08/H0718/76 and 06/13/LO/0751), and the Research Ethics Committee, Department of Psychological Sciences, Birkbeck, University of London.

## Supporting information


**Data S1:** Supporting Information.

## Data Availability

The data that support the findings of this study are available on request from the corresponding author. The data are not publicly available due to privacy or ethical restrictions.

## Footnotes

^1^
The ‘autism’ refers to Autism Spectrum Disorder (ASD; DSM‐5 (American Psychiatric Association., 2013)) and we also use ‘autistic individuals’ as this is a term widely accepted by the autism community (Kenny et al., 2016).
