# Supplementary material for: Associations between early language, motor abilities, and later autism traits in infants with typical and elevated likelihood of autism
Source: Autism Res. 2023 Sep 12;16(11):2184–97. doi: 10.1002/aur.3023 (PMC10899446; doi:10.1002/aur.3023)

**A prospective study of associations between early language, motor abilities and later autism traits in infants with typical and elevated likelihood of autism.**

Leyan Li MRes^1^, Greg Pasco PhD^1^, Jannath Begum Ali PhD^2^, Mark H. Johnson PhD^2,3^, Emily. J. H. Jones PhD^2^, Tony Charman PhD^1^

**Short title: Association between Language, Motor abilities and Autism traits.**

**Affiliations:** ^1^Institute of Psychiatry, Psychology & Neuroscience, King's College London, UK, ^2^Birkbeck, University of London, UK, ^3^University of Cambridge, UK

**Correspondence:** Tony Charman, Department of Psychology, Institute of Psychiatry, Psychology & Neuroscience, King’s College London, PO77, De Crespigny Park, Denmark Hill, London, SE5 8AF, UK; E-mail: [tony.charman@kcl.ac.uk](mailto:tony.charman@kcl.ac.uk)

**Table of Contents**

Supplementary Information of standardized measures used in the BASIS/STAARS

Supplementary Table 1: Missing data pattern

Supplementary Table 2: Correlations between the same type of Vineland and Mullen sub-scale scores

Supplementary Table 3: SRS and Sub-scales Correlations

Supplementary Table 4: Correlations between longitudinal data by group.

Supplementary Table 5: Confirmatory Factor Analysis of Language and Motor Skills across Four Time-points

Supplementary Figure 1: Structure of latent factors in CFA

Supplementary Figure 2: Comparison between original and cube-root transformed data

Supplementary Figure 3: Multi-group FM & EL RI-CLPM model excluding ASD cases

Supplementary Table 6: Linear Regression when taking out autism cases for FM-EL model

Supplementary Table 7: Linear Regression when taking out autism cases for GM-RL model

Supplementary Figure 4: Trible factor FM-EL-VR RI-CLPM with the outcome variable

Supplementary Figure 5: GM & EL uni-group RI-CLPM

Supplementary Figure 6: FM & RL uni-group RI-CLPM

**Supplementary Information of BASIS/STAARS standardised measures**

The term infants with ‘Elevated likelihood’ is used to define infants with autistic siblings who may retain a higher possibility to be diagnosed with ASD since they possess a broader genetic/familial heritability than their autistic siblings. ‘Typical likelihood’ is the term to define infants without 1st-degree family members with autism (controls), as they have comparatively low or even no possibility to be diagnosed with ASD.

As part of the British Autism Study of Infant Siblings (BASIS: http://www.basisnetwork.org), 247 infants were assessed four times, with visits at approximately 6–9, 12–15, 24 and 36 months of age. Infants in the EL group (n = 170; 85 males; 85 females) had at least one older sibling with a community clinical diagnosis of autism, which was confirmed based on parent reports: using the Development and Wellbeing Assessment (DAWBA; Goodman et al., 2000), the Social Communication Questionnaire (SCQ; Berument et al., 1999) or parent confirmed community clinical autism diagnosis. Infants in the TL group (n = 77; 35 male and 42 female) had at least one older sibling with typical development and no known autism in first-degree family members (as confirmed through parent interviews regarding family medical history). The Mullen Scales of Early Learning (MSEL; Mullen, 1995) and Vineland Adaptive Behaviour Scale-II (VABS-II; Sparrow et al., 2005) were administered at each visit. All toddlers were assessed at 24 and 36 months with the Autism Diagnostic Observation Schedule-2 (ADOS-2; Lord et al., 2012), and at 36 months, parents were interviewed using the Autism Diagnostic Interview-Revised (ADI-R; Lord et al., 1994). The best estimated clinical diagnosis of autism was made at age 3 informed by, but not dependent on outcomes from the ADOS-2, the ADI-R, the VABS-II and MSEL scores by experienced researchers (T.C. and G.P.). Thirty-four EL infants met the diagnostic criteria for autism at 36 months (see Supplementary Table S1). Participants were recruited from a volunteer database at the Birkbeck Centre for Brain and Cognitive Development. All parents included in the study completed written informed consent before each visit.

**Supplementary Table 1: Missing data pattern**

| **Time**  **Frequencies** | **Some data at 10 months** | | **Missing data at 10 months** | **Some data at 14 months** | | **Missing data at 14 months** | **Some data at 24 months** | **Missing data at 24 months** | **Some data at 36 months** | **Missing data at 36 months** |
| --- | --- | --- | --- | --- | --- | --- | --- | --- | --- | --- |
| **Vineland FM** | 370 | 38 (9.3%) | | 360 | 48 (11.8%) | | 356 | 52 (12.7%) | 345 | 63 (15.4%) |
| **Vineland GM** | 371 | 37 (9.1%) | | 363 | 45 (11.0%) | | 356 | 52 (12.7%) | 344 | 64 (15.7%) |
| **Mullen EL** | 395 | 13 (3.2%) | | 380 | 28 (7.0%) | | 356 | 52 (12.7%) | 365 | 52 (12.7%) |
| **Mullen RL** | 395 | 13 (3.2%) | | 380 | 28 (7.0%) | | 352 | 56 (13.7%) | 356 | 52 (12.4%) |
| **SRS total** |  | | | | | | | | 332 | 76 (18.6%) |

**Note:** A total of 408 children participated in an intake assessment, and 395 completed either the Vineland or the Mullen (or both) at 10 months. The percentage of all missing values was shown in brackets. The SRS-total score was only measured at 36 months.

Vineland FM = Vineland Fine Motor; Vineland GM = Vineland Gross Motor; Mullen EL = Mullen Expressive Language; Mullen RL= Mullen Receptive Language; SRS total = Social Responsiveness Scales Total Scores at 36 months.

**Supplementary Table 2: Correlations between the same type of Vineland and Mullen sub-scale scores**

| Variables | MFM at 10m | MGM at 10m | VFM at 10m | VGM at 10m | MFM at 14m | MGM at 14m | VFM at 14m | VGM at 14m | MFM at 24m | VFM at 24m | VGM at 24m | MFM at 36m | VFM at 36m | VGM at 36m |
| --- | --- | --- | --- | --- | --- | --- | --- | --- | --- | --- | --- | --- | --- | --- |
| Mullen Fine Motor at 10 m | -- |  |  |  |  |  |  |  |  |  |  |  |  |  |
| Mullen Gross Motor at 10 m | **.487**** | -- |  |  |  |  |  |  |  |  |  |  |  |  |
| Vineland Fine Motor at 10 m | **.466**** | **.427**** | -- |  |  |  |  |  |  |  |  |  |  |  |
| Vineland Gross Motor at 10 m | **.516**** | **.761**** | **.575**** | -- |  |  |  |  |  |  |  |  |  |  |
| Mullen Fine Motor at 14 m | **.299**** | **.281**** | **.203**** | **.138**** | -- |  |  |  |  |  |  |  |  |  |
| Mullen Gross Motor at 14 m | **.324**** | **.487**** | **.219**** | **.428**** | **.351**** | -- |  |  |  |  |  |  |  |  |
| Vineland Fine Motor at 14 m | **.264**** | **.289**** | **.415**** | **.300**** | **.325**** | **.332**** | -- |  |  |  |  |  |  |  |
| Vineland Gross Motor at 14 m | **.371**** | **.589**** | **.372**** | **.561**** | **.298**** | **.810**** | **.468**** | -- |  |  |  |  |  |  |
| Mullen Fine Motor at 24 m | **.298**** | **.152**** | **.254**** | **.211**** | **.315**** | **.190**** | **.226**** | **.232**** | -- |  |  |  |  |  |
| Vineland Fine Motor at 24 m | **.126*** | .078 | **.166**** | .057 | **.327**** | **.206**** | **.413**** | **.231**** | **.382**** | -- |  |  |  |  |
| Vineland Gross Motor at 24 m | **.303**** | .**372**** | **.313**** | **.370**** | **.249**** | **.433**** | **.403**** | **.500**** | **.337**** | **.438**** | -- |  |  |  |
| Mullen Fine Motor at 36 m | **.271**** | .095 | **.158**** | .094 | **.349**** | **.132*** | **.185**** | **.160**** | **.498**** | **.362**** | **.233**** | -- |  |  |
| Vineland Fine Motor at 36 m | .106 | **.127**** | .094 | .041 | **.291**** | **.199**** | **.216**** | **.165**** | **.302**** | **.451**** | **.245**** | **.503**** | -- |  |
| Vineland Gross Motor at 36 m | .099 | **.158**** | **.169**** | .109 | **.216**** | **.203**** | **.229**** | **.204**** | **.305**** | **.256**** | **.378**** | **.369**** | **.514**** | **--** |
| SRS total at 36 m | **-.112*** | .002 | -.077 | .007 | **-.222**** | .033 | **-.154**** | .029 | **-.261**** | **-.217**** | **-.181**** | **-.362**** | **-.282**** | **-.282**** |
| **. Correlation is significant at the 0.01 level (2-tailed). | | | | | | | | | | | | | | |
| *. Correlation is significant at the 0.05 level (2-tailed).  Note: 24- & 36-months Mullen gross motor measures are not available. Cross-method associations are highlighted. | | | | | | | | | | | | | | |

***Correlations for Vineland and Mullen motor scores***

| Variables | | | MEL at 10m | MRL at 10m | VEL at 10m | VRL at 10m | MEL at 14m | MRL at 14m | VEL at 10m | VRL at 10m | MEL at 24m | MRL at 24m | VEL at 10m | VRL at 10m | MEL at 36m | MRL at 36m | VEL at 10m | VRL at 10m |
| --- | --- | --- | --- | --- | --- | --- | --- | --- | --- | --- | --- | --- | --- | --- | --- | --- | --- | --- |
| Mullen Expressive Language at 10 m | | | -- |  |  |  |  |  |  |  |  |  |  |  |  |  |  |  |
| Mullen Receptive Language at 10 m | | | **.493**** | -- |  |  |  |  |  |  |  |  |  |  |  |  |  |  |
| Vineland Expressive Language at 10 m | | | **.359**** | **.294**** | -- |  |  |  |  |  |  |  |  |  |  |  |  |  |
| Vineland Receptive Language at 10 m | | | **.165**** | **.311**** | **.517**** | -- |  |  |  |  |  |  |  |  |  |  |  |  |
| Mullen Expressive Language at 14 m | | | **.450**** | **.340**** | **.249**** | **.149**** | -- |  |  |  |  |  |  |  |  |  |  |  |
| Mullen Receptive Language at 14 m | | | **.361**** | **.304**** | **.144**** | .096 | **.627**** | -- |  |  |  |  |  |  |  |  |  |  |
| Vineland Expressive Language at 14 m | | | **.342**** | **.326**** | **.503**** | **.358**** | **.641**** | **.491**** | -- |  |  |  |  |  |  |  |  |  |
| Vineland Receptive Language at 14 m | | | **.297**** | **.350**** | **.365**** | **.380**** | **.532**** | **.582**** | **.692**** | -- |  |  |  |  |  |  |  |  |
| Mullen Expressive Language at 24 m | | | **.214**** | **.257**** | **.188**** | **.178**** | **.425**** | **.410**** | **.427**** | **.394**** | -- |  |  |  |  |  |  |  |
| Mullen Receptive Language at 24 m | | | **.142**** | **.240**** | **.179**** | **.199**** | **.388**** | **.377**** | **.436**** | **.412**** | **.697**** | -- |  |  |  |  |  |  |
| Vineland Expressive Language at 24 m | | | **.190**** | **.275**** | **.265**** | **.260**** | **.408**** | **.361**** | **.524**** | **.472**** | **.799**** | **.678**** | -- |  |  |  |  |  |
| Vineland Receptive Language at 24 m | | | **.121*** | **.228**** | **.211**** | **.245**** | **.287**** | **.268**** | **.423**** | **.447**** | **.546**** | **.649**** | **.687**** | -- |  |  |  |  |
| Mullen Expressive Language at 36 m | | | **.117*** | **.201**** | **.113**** | **.112*** | **.315**** | **.362**** | **.363**** | **.342**** | **.640**** | **.613**** | **.591**** | **.537**** | -- |  |  |  |
| Mullen Receptive Language at 36m | | | .052 | **.172**** | **.153**** | **.140**** | **.233**** | **.249**** | **.286**** | **.284**** | **.576**** | **.626**** | **.567**** | **.585**** | **.785**** | -- |  |  |
| Vineland Expressive Language at 36 m | | | .092 | **.193**** | **.155**** | **.179**** | **.344**** | **.332**** | **.412**** | **.396**** | **.647**** | **.659**** | **.650**** | **.635**** | **.765**** | **.766**** | -- |  |
| Vineland Receptive Language at 36 m | | | **.145**** | **.202**** | **.140**** | **.125*** | **.378**** | **.324**** | **.371**** | **.389**** | **.565**** | **.625**** | **.610**** | **.647**** | **.673**** | **.679**** | **.791**** | -- |
| SRS total at 36m | | | -.007 | **-**.089 | **-.119*** | **-.167**** | **-.269**** | **-.188**** | **-.310**** | **-.287**** | **-.287**** | **-.436**** | **-.381**** | **-.462**** | **-.350**** | **-.352**** | **-.498**** | **-.522**** |
|  |  | **. Correlation is significant at the 0.01 level (2-tailed). | | | | | | | | | | | | | | | | |
|  |  | *. Correlation is significant at the 0.05 level (2-tailed).  Note: 24- & 36-month Mullen gross motor measures are not available. Cross-method associations are highlighted. | | | | | | | | | | | | | | | | |

***Correlations for Vineland and Mullen Language Score***

**Supplementary Table 3: SRS and Sub-scales Correlations**

**Overall**

| **Time** | **SRS** | **EL-Mullen** | **EL-Vineland** | **RL-Mullen** | **RL-Vineland** | **FM-Mullen** | **FM-Vineland** | **GM-Mullen** | **GM-Vineland** |
| --- | --- | --- | --- | --- | --- | --- | --- | --- | --- |
| **10 m** |  | -.001 | **-.119^*^** | -.089 | **-.167^**^** | **-.112*** | -.077 | .002 | .007 |
| **14 m** |  | **-.268^**^** | **-.310^**^** | **-.188^**^** | **-.287^**^** | **-.222^**^** | **-.154^**^** | .033 | .029 |
| **24 m** |  | **-.287^**^** | **-.381^**^** | **-.436^**^** | **-.462^**^** | **-.261^**^** | **-.217^**^** | N/A | **-.181^**^** |
| **36 m** |  | **-.350^**^** | **-.498^**^** | **-.352^**^** | **-.522^**^** | **-.326^**^** | **-.282^**^** | N/A | **-.282^**^** |

**Elevated Likelihood Group**

| **Time** | **SRS** | **EL-Mullen** | **EL-Vineland** | **RL-Mullen** | **RL-Vineland** | **GM-Mullen** | **GM-Vineland** |
| --- | --- | --- | --- | --- | --- | --- | --- |
| **14 m** |  | **-.272^**^** | **-.310^**^** |  | | | |
| **24 m** |  |  | | **-.408^**^** | **-.447^**^** |  | |
| **36 m** |  |  | | | | N/A | **-.289^**^** |

**Note:** Numbers indicate Pearson correlation coefficients
those in bold indicates significance, *indicates a significant level at .005 and ** indicates a significant level at .001.
Numbers highlighted are identified factors by the Random Intercept Cross-Lagged Panel model.
N/A indicates variables unavailable.

| ***Correlations for Typical likelihood group*** | | | | | | | | | | | | | | | | |
| --- | --- | --- | --- | --- | --- | --- | --- | --- | --- | --- | --- | --- | --- | --- | --- | --- |
| Variables | FM at 10m | FM at 14m | FM at 24m | FM at 36m | EL at 10m | EL at 14m | EL at 24m | EL at 36m | GM at 10m | GM at 14m | GM at 24m | GM at 36m | RL at 10m | RL at 14m | RL at 24m | RL at 36m |
| Fine Motor at 10 m | -- |  |  |  |  |  |  |  |  |  |  |  |  |  |  |  |
| Fine Motor at 14 m | .**257**** | -- |  |  |  |  |  |  |  |  |  |  |  |  |  |  |
| Fine Motor at 24 m | .062 | **.329**** | -- |  |  |  |  |  |  |  |  |  |  |  |  |  |
| Fine Motor at 36 m | -.011 | .161 | **.450**** | -- |  |  |  |  |  |  |  |  |  |  |  |  |
| Expressive Language at 10 m | .093 | .**229*** | .091 | .156 | -- |  |  |  |  |  |  |  |  |  |  |  |
| Expressive Language at 14 m | .011 | **.264**** | **.190*** | **.312**** | **.484**** | -- |  |  |  |  |  |  |  |  |  |  |
| Expressive Language at 24 m | .145 | .139 | **.329**** | **.334**** | **.201*** | **.371**** | -- |  |  |  |  |  |  |  |  |  |
| Expressive Language at 36 m | .027 | .017 | **.328**** | **.340**** | **.138** | **.181** | **.522**** | -- |  |  |  |  |  |  |  |  |
| Gross Motor at 10 m | **.531**** | .024 | -.026 | -.102 | .038 | -.025 | .099 | .039 | -- |  |  |  |  |  |  |  |
| Gross Motor at 14 m | **.285**** | **.337**** | .117 | .169 | **.205*** | **.270**** | .128 | .028 | **.491**** | -- |  |  |  |  |  |  |
| Gross Motor at 24 m | .148 | **.288**** | .**320**** | .101 | .**212*** | **.211*** | **.219*** | .020 | **.306**** | **.418**** | -- |  |  |  |  |  |
| Gross Motor at 36 m | .015 | .151 | .379** | .**339**** | .036 | .125 | .100 | .146 | -.091 | .101 | **.233*** | -- |  |  |  |  |
| Receptive Language at 10 m | **.305**** | .120 | .088 | .006 | **.431**** | **.372**** | **.259**** | .171 | **.211*** | **.194*** | .179 | -.166 | -- |  |  |  |
| Receptive Language at 14 m | .023 | **.248**** | .100 | **.313**** | **.449**** | **.624**** | **.340**** | **.338**** | -.081 | .133 | .061 | .106 | **.322**** | **--** |  |  |
| Receptive Language at 24 m | .077 | .062 | **.269**** | .150 | .149 | **.257**** | **.519**** | **.606**** | .009 | -.011 | .044 | .058 | **.272**** | **.345**** | -- |  |
| Receptive Language at 36 m | .088 | -.053 | **.362**** | **.200*** | .034 | .064 | **.373**** | **.639**** | .086 | -.132 | .001 | .088 | .182 | .124 | **.531**** | -- |
| **. Correlation is significant at the 0.01 level (2-tailed). | | | | | | | | | | | | | | | | |
| *. Correlation is significant at the 0.05 level (2-tailed).  Note: Motor measures are provided by Vineland scales and language measures from Mullen Scales. | | | | | | | | | | | | | | | | |

**Supplementary Table 4: Correlations between latent factors by groups**

| ***Correlations for Elevated likelihood group*** | | | | | | | | | | | | | | | | |
| --- | --- | --- | --- | --- | --- | --- | --- | --- | --- | --- | --- | --- | --- | --- | --- | --- |
| Variables | FM at 10m | FM at 14m | FM at 24m | FM at 36m | EL at 10m | EL at 14m | EL at 24m | EL at 36m | GM at 10m | GM at 14m | GM at 24m | GM at 36m | RL at 10m | RL at 14m | RL at 24m | RL at 36m |
| Fine Motor at 10 m | -- |  |  |  |  |  |  |  |  |  |  |  |  |  |  |  |
| Fine Motor at 14 m | **.450**** | **--** |  |  |  |  |  |  |  |  |  |  |  |  |  |  |
| Fine Motor at 24 m | **.184**** | **.425**** | **--** |  |  |  |  |  |  |  |  |  |  |  |  |  |
| Fine Motor at 36 m | .085 | **.182**** | **.436**** | **--** |  |  |  |  |  |  |  |  |  |  |  |  |
| Expressive Language at 10 m | **.227**** | **.259**** | **.141*** | .025 | **--** |  |  |  |  |  |  |  |  |  |  |  |
| Expressive Language at 14 m | **.173**** | **.337**** | **.306**** | **.264**** | **.440**** | **--** |  |  |  |  |  |  |  |  |  |  |
| Expressive Language at 24 m | **.172**** | **.260**** | **.419**** | **.354**** | **.227**** | **.429**** | **--** |  |  |  |  |  |  |  |  |  |
| Expressive Language at 36 m | .063 | **.177**** | **.299**** | **.459**** | .125 | **.335**** | **.651**** | **--** |  |  |  |  |  |  |  |  |
| Gross Motor at 10 m | **.585**** | **.391**** | **.**078 | .068 | **.226**** | .044 | .087 | -.002 | **--** |  |  |  |  |  |  |  |
| Gross Motor at 14 m | **.401**** | **.517**** | **.269**** | **.148*** | **.280**** | **.300**** | **.147*** | .065 | **.592**** | **--** |  |  |  |  |  |  |
| Gross Motor at 24 m | **.355**** | **.427**** | **.476**** | **.282**** | **.213**** | **.251**** | **.310**** | **.231**** | **.388**** | **.527**** | **--** |  |  |  |  |  |
| Gross Motor at 36 m | **.188**** | **.215**** | **.197**** | **.561**** | .040 | **.216**** | **.200**** | **.358**** | **.161*** | **.227**** | **.410**** | **--** |  |  |  |  |
| Receptive Language at 10 m | **.374**** | **.279**** | **.135*** | **.149*** | **.517**** | **.338**** | **.276**** | **.239**** | **.294**** | **.303**** | **.221**** | **.155*** | **--** |  |  |  |
| Receptive Language at 14 m | .080 | **.302**** | **.319**** | **.272**** | **.327**** | **.628**** | **.429**** | **.364**** | .020 | **.262**** | **.235**** | **.225**** | **.307**** | **--** |  |  |
| Receptive Language at 24 m | **.160*** | **.230**** | **.442**** | **.382**** | **.147*** | **.412**** | **.736**** | **.583**** | **.141*** | **.174**** | **.368**** | **.216**** | **.250**** | **.382**** | **--** |  |
| Receptive Language at 36 m | **.148*** | **.139*** | **.280**** | **.406**** | .069 | **.258**** | **.604**** | **.802**** | .074 | .049 | **.200**** | **.353**** | **.197**** | **.276**** | **.617**** | -- |
| **. Correlation is significant at the 0.01 level (2-tailed). | | | | | | | | | | | | | | | | |
| *. Correlation is significant at the 0.05 level (2-tailed).  Note: Motor measures are provided by Vineland scales and language measures from Mullen Scales. | | | | | | | | | | | | | | | | |

| ***Correlations for Elevated Likelihood Group without Autism Outcome*** | | | | | | | | | | | | | | | | |
| --- | --- | --- | --- | --- | --- | --- | --- | --- | --- | --- | --- | --- | --- | --- | --- | --- |
| Variables | FM at 10m | FM at 14m | FM at 24m | FM at 36m | EL at 10m | EL at 14m | EL at 24m | EL at 36m | GM at 10m | GM at 14m | GM at 24m | GM at 36m | RL at 10m | RL at 14m | RL at 24m | RL at 36m |
| Fine Motor at 10 m | -- |  |  |  |  |  |  |  |  |  |  |  |  |  |  |  |
| Fine Motor at 14 m | **.438**** | -- |  |  |  |  |  |  |  |  |  |  |  |  |  |  |
| Fine Motor at 24 m | **.204**** | **.399**** | -- |  |  |  |  |  |  |  |  |  |  |  |  |  |
| Fine Motor at 36 m | .070 | **.170*** | **.455**** | -- |  |  |  |  |  |  |  |  |  |  |  |  |
| Expressive Language at 10 m | .105 | **.191**** | .128 | .026 | -- |  |  |  |  |  |  |  |  |  |  |  |
| Expressive Language at 14 m | **.150*** | **.309**** | **.267**** | **.289**** | **.456**** | -- |  |  |  |  |  |  |  |  |  |  |
| Expressive Language at 24 m | **.216**** | **.274**** | **.413**** | **.334**** | **.251**** | **.390**** | -- |  |  |  |  |  |  |  |  |  |
| Expressive Language at 36 m | .091 | **.187**** | **.309**** | **.442**** | .128 | **.267**** | **.612**** | -- |  |  |  |  |  |  |  |  |
| Gross Motor at 10 m | **.605**** | **.335**** | .071 | .038 | .129 | .010 | .117 | .061 | -- |  |  |  |  |  |  |  |
| Gross Motor at 14 m | **.393**** | **.543**** | **.325**** | **.155*** | **.295**** | **.328**** | **.230**** | **.177*** | **.536**** | -- |  |  |  |  |  |  |
| Gross Motor at 24 m | **.374**** | **.427**** | **.486**** | **.270**** | **.200**** | **.230**** | **.289**** | **.219**** | **.379**** | **.531**** | -- |  |  |  |  |  |
| Gross Motor at 36 m | **.180*** | **.247**** | **.264**** | **.588**** | .039 | **.287**** | **.180**** | **.286**** | .131 | **.256**** | **.428**** | -- |  |  |  |  |
| Receptive Language at 10 m | **.371**** | **.259**** | .159* | .091 | **.454**** | **.328**** | **.275**** | **.232**** | **.219**** | **.304**** | **.222**** | .070 | -- |  |  |  |
| Receptive Language at 14 m | .061 | **.321**** | **.305**** | **.282**** | **.365**** | **.621**** | **.423**** | **.344**** | -.029 | **.291**** | **.220**** | **.237**** | **.305**** | -- |  |  |
| Receptive Language at 24 m | **.142*** | **.239**** | **.437**** | **.368**** | **.161*** | **.380**** | **.715**** | **.551**** | .093 | .219** | .315** | .170* | .229** | .368** | -- |  |
| Receptive Language at 36 m | **.211**** | **.166*** | **.318**** | **.397**** | .050 | **.215**** | **.579**** | **.747**** | **.168*** | **.176*** | **.204**** | **.210**** | **.176**** | **.255**** | **.580**** | -- |
| **. Correlation is significant at the 0.01 level (2-tailed).* | | | | | | | | | | | | | | | | |
| *. Correlation is significant at the 0.05 level (2-tailed).  Note: Motor measures are provided by Vineland scales and language measures from Mullen Scales. | | | | | | | | | | | | | | | | |

| ***Correlations for Elevated likelihood group with Autism Outcome*** | | | | | | | | | | | | | | | | |
| --- | --- | --- | --- | --- | --- | --- | --- | --- | --- | --- | --- | --- | --- | --- | --- | --- |
| Variables | FM at 10m | FM at 14m | FM at 24m | FM at 36m | EL at 10m | EL at 14m | EL at 24m | EL at 36m | GM at 10m | GM at 14m | GM at 24m | GM at 36m | RL at 10m | RL at 14m | RL at 24m | RL at 36m |
| Fine Motor at 10 m | -- |  |  |  |  |  |  |  |  |  |  |  |  |  |  |  |
| Fine Motor at 14 m | **.549**** | -- |  |  |  |  |  |  |  |  |  |  |  |  |  |  |
| Fine Motor at 24 m | .112 | **.556**** | -- |  |  |  |  |  |  |  |  |  |  |  |  |  |
| Fine Motor at 36 m | .144 | .249 | **.520**** | -- |  |  |  |  |  |  |  |  |  |  |  |  |
| Expressive Language at 10 m | **.482**** | **.424**** | .037 | .055 | -- |  |  |  |  |  |  |  |  |  |  |  |
| Expressive Language at 14 m | .222 | **.479**** | **.385*** | .121 | **.567**** | -- |  |  |  |  |  |  |  |  |  |  |
| Expressive Language at 24 m | .045 | **.318*** | **.584**** | **.401**** | .107 | **.472**** | -- |  |  |  |  |  |  |  |  |  |
| Expressive Language at 36 m | .021 | .166 | **.311*** | **.451**** | .152 | **.329*** | **.746**** | -- |  |  |  |  |  |  |  |  |
| Gross Motor at 10 m | **.514**** | **.484**** | .137 | .249 | **.327*** | .009 | -.050 | -.088 | -- |  |  |  |  |  |  |  |
| Gross Motor at 14 m | **.448**** | **.448**** | .166 | .242 | .281 | .159 | -.065 | -.101 | **.815**** | -- |  |  |  |  |  |  |
| Gross Motor at 24 m | .228 | **.454**** | **.492**** | **.375*** | .105 | .297 | **.382*** | .295 | **.355*** | **.481**** | -- |  |  |  |  |  |
| Gross Motor at 36 m | .061 | .058 | -.045 | **.369*** | -.052 | -.178 | .081 | **.317*** | .182 | .190 | .288 | -- |  |  |  |  |
| Receptive Language at 10 m | **.441**** | **.422**** | .118 | .240 | **.599**** | **.383**** | .233 | .160 | **.426**** | **.391**** | .165 | .059 | -- |  |  |  |
| Receptive Language at 14 m | .247 | **.453**** | **.315*** | .197 | **.301*** | **.652**** | **.415**** | .213 | .094 | .218 | .280 | .007 | **.369*** | -- |  |  |
| Receptive Language at 24 m | .136 | **.340*** | **.584**** | **.349*** | .094 | **.408**** | **.732**** | **.559**** | .151 | .113 | **.528**** | .054 | .172 | **.392**** | -- |  |
| Receptive Language at 36 m | .080 | .109 | .268 | **.375*** | .134 | .207 | **.697**** | **.844**** | .012 | -.083 | .246 | **.444**** | .184 | .175 | **.634**** | -- |
| **. Correlation is significant at the 0.01 level (2-tailed).* | | | | | | | | | | | | | | | | |
| *. Correlation is significant at the 0.05 level (2-tailed).  Note: Motor measures are provided by Vineland scales and language measures from Mullen Scales. | | | | | | | | | | | | | | | | |

**Supplementary Table 5: Confirmatory Factor Analysis of Language and Motor Skills across Four Time-points**

| **Items** | **10m**  **Factor Loadings/R^2^** | **14m**  **Factor Loadings/ R^2^** | **24m**  **Factor Loadings/ R^2^** | **36m**  **Factor Loadings/ R^2^** |
| --- | --- | --- | --- | --- |
| **Model 1** | | | | |
| Expressive language | 1.00 / 1.00 | 1.00 / 1.00 | 1.00 / 1.00 | 1.00 / 1.00 |
| Fine motor | 1.00 / 1.00 | 1.00 / 1.00 | 1.00 / 1.00 | 1.00 / 1.00 |
| **Fit indices** | | | | |
| CFI | 1.000 | | | |
| RMSEA | < .000 | | | |
| SRMR | < .000 | | | |
| **Model 2** | | | | |
| Receptive language | 1.00 / 1.00 | 1.00 / 1.00 | 1.00 / 1.00 | 1.00 / 1.00 |
| Gross motor | 1.00 / 1.00 | 1.00 / 1.00 | 1.00 / 1.00 | 1.00 / 1.00 |
| **Fit indices** | | | | |
| CFI | 1.000 | | | |
| RMSEA | < .000 | | | |
| SRMR | < .000 | | | |

**Note:** Standardized factor loadings and model estimates are presented. Confirmatory factor analysis supported subscales of language and motor skills as separating into two factors at each time point. All factor loadings were greater than 0.8, and variables’ R^2^ were greater than 0.8 and major indicators of model estimation were all above good level.

**Supplementary Figure 1: Structure of latent factors in CFA**


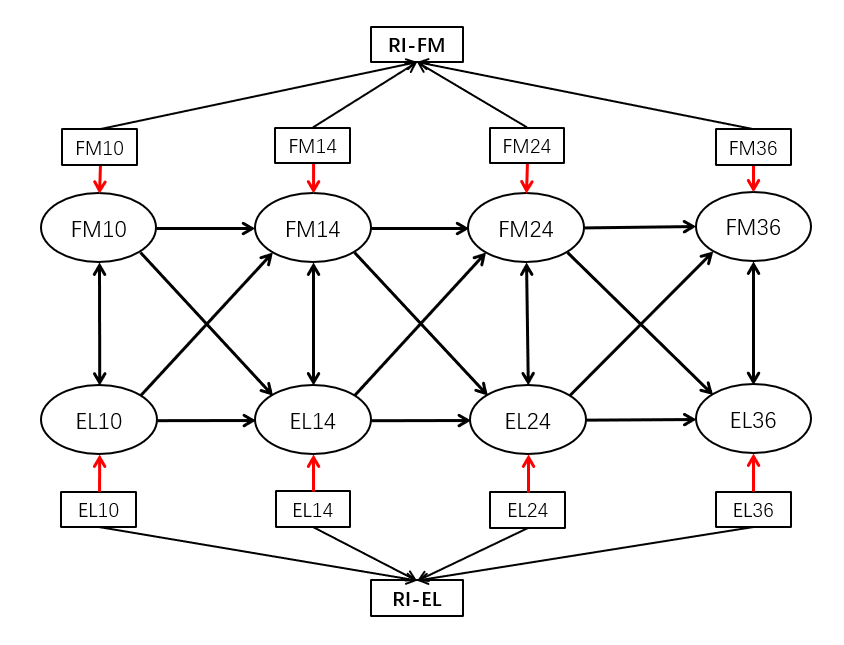


1. Fine motor and Expressive language Random Intercept Cross-Lagged Panel Model


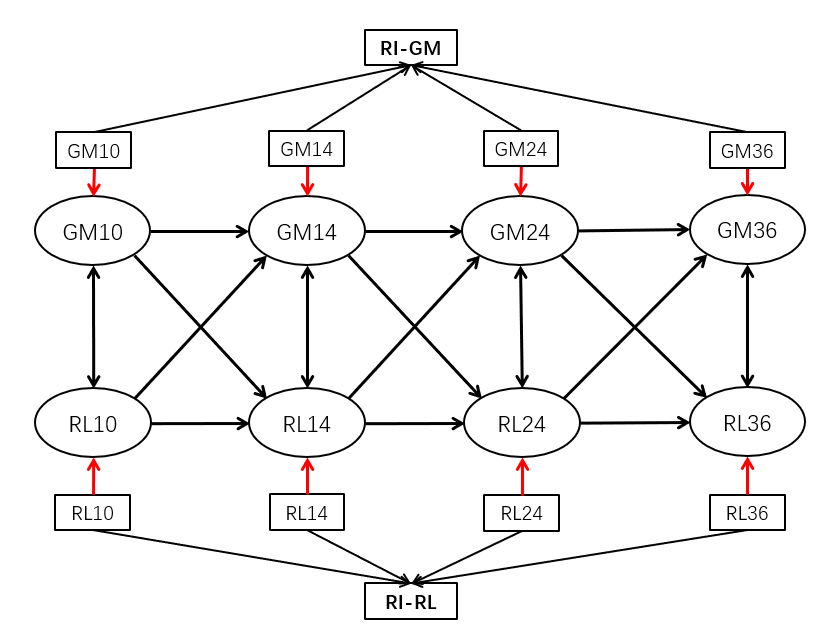


1. Gross motor and Receptive language Random Intercept Cross-Lagged Panel Model

**Supplementary Figure 2: Comparison between original data and cube-root transformed data**


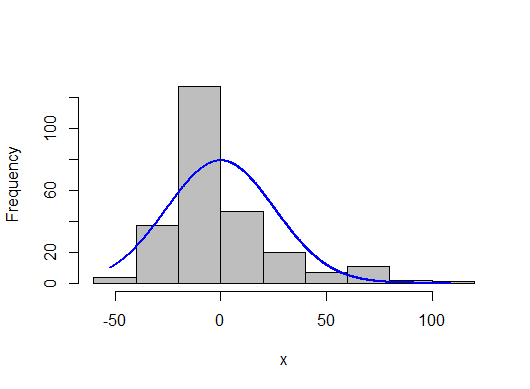

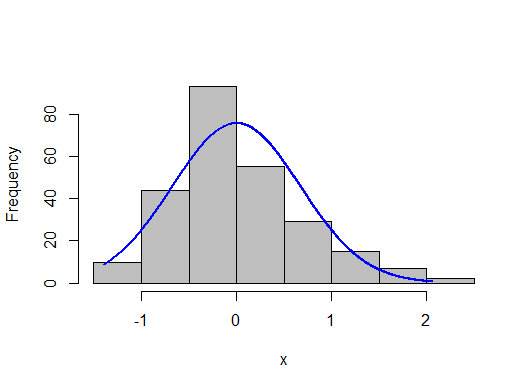


(1). Distribution of Original data (2). Distribution of Cube root transformed data


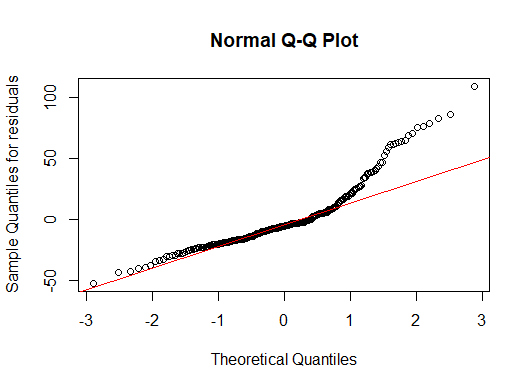

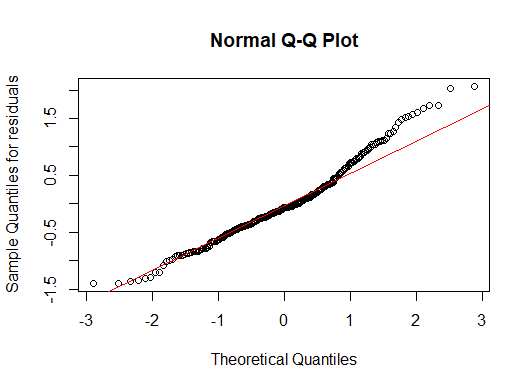


(3). Q-Q plot of original data (4). Q-Q plot of Cube root transformed data


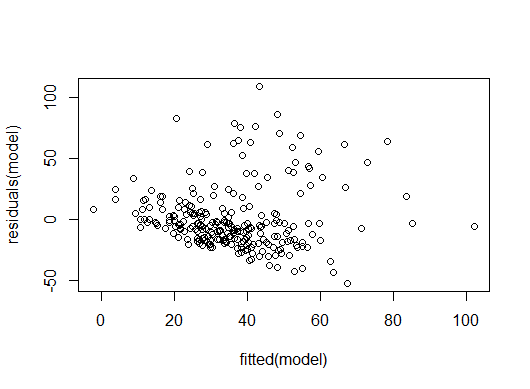

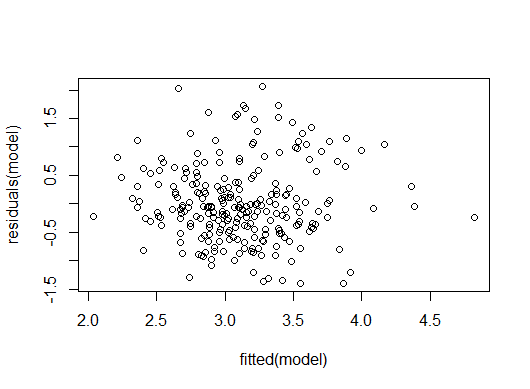


(5). Residual model of original data (6). Residual model of Cube root transformed data

**Supplementary Figure 3: Multi-group FM & EL RI-CLPM model excluding ASD cases**


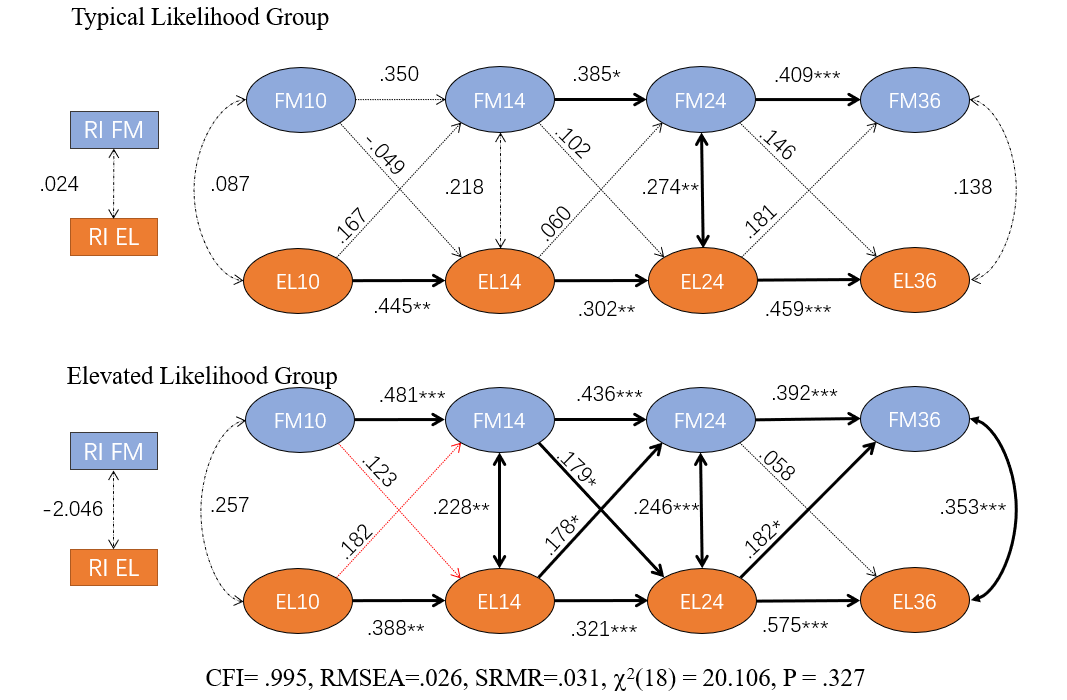


**Supplementary Table 6: Linear Regression when taking out autism cases for FM-EL model**

*Regression 1: 14 months Expressive language and 36 months SRS-total score in Elevated likelihood group*

| Effect | Estimate | *SE* | 95% CI | | *p* |
| --- | --- | --- | --- | --- | --- |
|  |  |  | *LL* | *UL* |  |
|  |  |  |  |  |  |
| Intercept |  | 8.824 | 60.498 | 95.287 | .001 |
| EL at 14 months | -.272 | .640 | -3.900 | -1.377 | .001 |

*Note. N* = 214. CI = confidence interval; *LL* = lower limit; *UL* = upper limit.

*Regression 2: 14 months Expressive language and 36 months SRS-total score in Elevated likelihood group without autism cases*

| Effect | Estimate | *SE* | 95% CI | | *p* |
| --- | --- | --- | --- | --- | --- |
|  |  |  | *LL* | *UL* |  |
|  |  |  |  |  |  |
| Intercept |  | 7.346 | 34.146 | 63.151 | .001 |
| EL at 14 months | -.170 | .519 | -2.191 | -.141 | .026 |

*Note. N* = 171. CI = confidence interval; *LL* = lower limit; *UL* = upper limit.

For 14 months EL predicting 36 months SRS total scores in the EL group only, the result of linear regression suggests that the 14m EL shows a significant relation to 36 months SRS in groups with or without the autism cases included in the sample (B *_overall_* =-.272, *p* <.001, t=-4.123; B *_autism excluded_* =-.170, *p _autism excluded_* =.026, t=-2.246). The model including the full Elevated likelihood group (271 participants) explained 7.4% of the total variance (R^2^= .074, F (1,213) = 16.997, *p*<.001) while the Elevated likelihood group with the autism cases excluded only accounts for 2.9% of the total variance (R^2^= .029, F (1,170) = 5.043, *p*=.026). Therefore, the autism cases (40 participants) contributed more than half of the explained variance.

**Supplementary Table 7: Linear Regression when taking out autism cases for GM-RL model**

*Regression 3: 24 months Receptive language, 36 months Gross motor and 36 months SRS-total score in Elevated likelihood group*

| Effect | Estimate | *SE* | 95% CI | | *p* |
| --- | --- | --- | --- | --- | --- |
|  |  |  | *LL* | *UL* |  |
|  |  |  |  |  |  |
| Intercept |  | 21.059 | 135.519 | 218.584 | .001 |
| RL at 24 months | -.351 | .451 | -3.332 | -1.554 | .001 |
| GM at 36 months | -.236 | .325 | -1.823 | -.542 | .001 |

*Note. N* = 197. CI = confidence interval; *LL* = lower limit; *UL* = upper limit.

*Regression 4: 24 months Receptive language, 36 months Gross motor and 36 months SRS-total score in Elevated likelihood group without autism cases*

| Effect | Estimate | *SE* | 95% CI | | *p* |
| --- | --- | --- | --- | --- | --- |
|  |  |  | *LL* | *UL* |  |
|  |  |  |  |  |  |
| Intercept |  | 19.918 | 66.053 | 144.739 | .001 |
| RL at 24 months | -.176 | .423 | -1.797 | -.125 | .025 |
| GM at 36 months | -.204 | .290 | -1.339 | -.191 | .009 |

*Note. N* = 158. CI = confidence interval; *LL* = lower limit; *UL* = upper limit.

For 24 months RL and 36 months GM predicting 36 months SRS total scores, the result of linear regression suggests that the 24m RL and 36m GM are significantly associated with 36 months SRS in models with or without autism cases (B *_24RL-overall_* =-.351, *p _24RL-overall_* <.001, t *_24RL-overall_* =-5.417; B *_24RL- autism excluded_* =-.176, *p _24RL_-_autism excluded_* =.025, t=-2.270; B *_36GM-overall_* =-.236, *p _36GM-overall_* <.001, t *_36GM-overall_* =-3.642; B *_36GM- autism excluded_* =-.204, *p _36GM_-_autism excluded_* =.009, t *_36GM_-_autism excluded_* =-2.634). Similarly, the model including the full Elevated likelihood group (271 participants) explained 21.3% of the total variance (R^2^= .213, F (2,197) = 26.395, *p*<.001) while the model excluding the autism cases only explained 8.4% of the total variance in SRS scores (R^2^= .084, F (2,158) = 7.118, *p*<.001). Therefore, the autism cases (40 participants) contributed more than half of the explained variance.

In conclusion, all the results above are in line with our inference that effects detected in these models were weaker when participants with autism are excluded, and hence capture processes that are meaningfully related to autism within the sample. However, the fact that dimensional relations between infant language/motor scores and later autistic traits remain significant in the EL group when the cases with autism are removed indicates that these relationships are also present at the trait level.

**Supplementary Figure 4: Triple Factor VFM-MEL-MVR RI-CLPM model with the outcome variable**


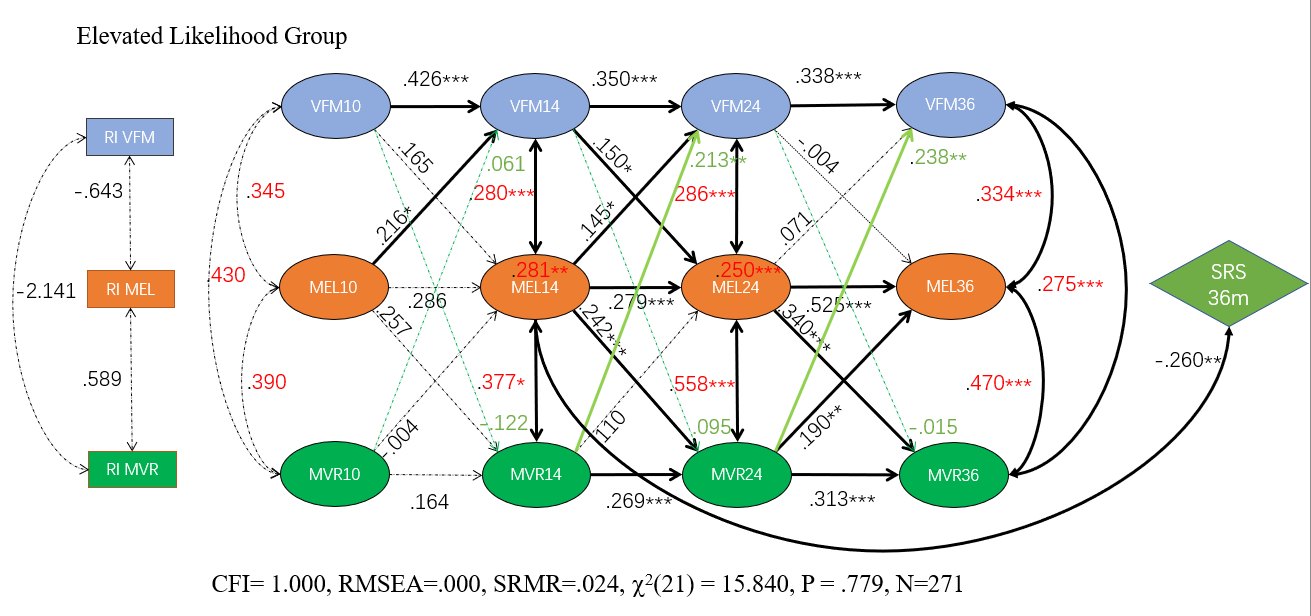


**Figure 4:** Triple factor Random Intercept Cross-lagged Panel model on Vineland Fine Motor, Mullen Expressive Language and Visual Reception in the Elevated Likelihood group with inclusion of the outcome variable SRS total.

**Note:** Numbers in red indicate between variable estimates. Numbers next to arrows and paths in green indicate cross-lagged effect and their estimates. Only significant path between latent and outcome variables are included.

**Supplementary Figure 5: Uni-group GM & EL RI-CLPM**


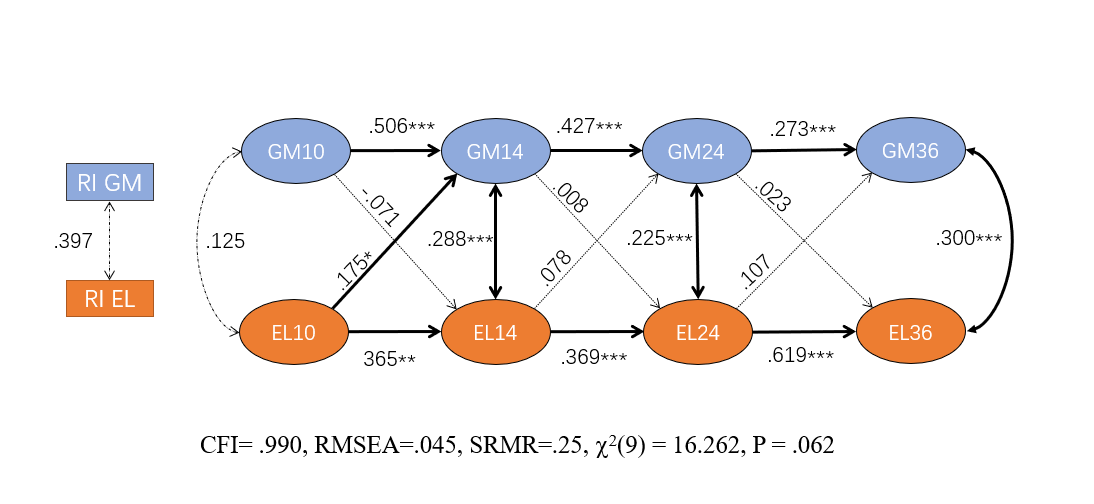


**Supplementary Figure 6: Uni-group FM & RL RI-CLPM**


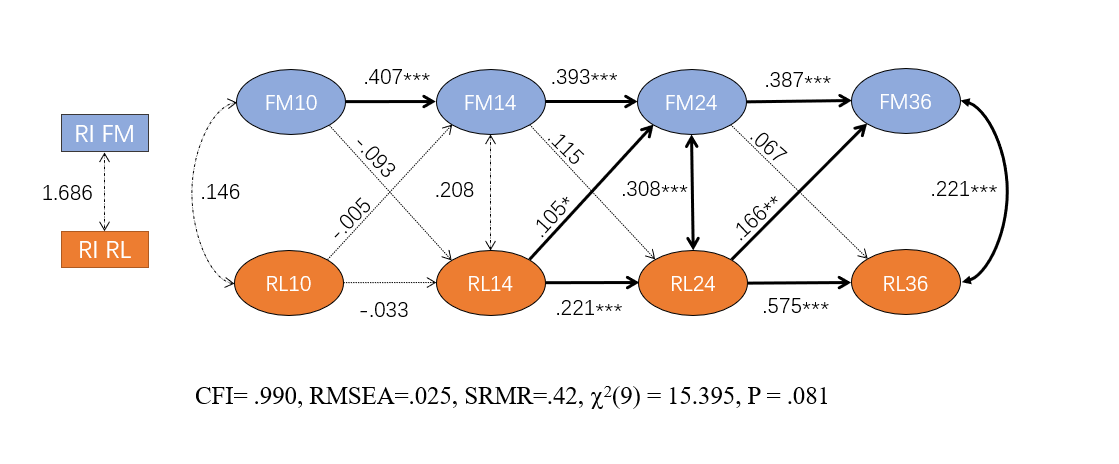

Supplement: Supplementary file 1 — Data S1: Supporting Information. [file AUR-16-2184-s001.docx]
